# The Nutritional and Safety Challenges Associated with Lupin Lacto-Fermentation

**DOI:** 10.3389/fpls.2016.00951

**Published:** 2016-06-28

**Authors:** Elena Bartkiene, Vadims Bartkevics, Vytaute Starkute, Daiva Zadeike, Grazina Juodeikiene

**Affiliations:** ^1^Food Safety and Quality, Lithuanian University of Health SciencesKaunas, Lithuania; ^2^Chemistry, BIORRyga, Latvia; ^3^Kaunas University of TechnologyKaunas, Lithuania

**Keywords:** lupins, fermentation, nutrition, safety, lactic acid bacteria

## Introduction

A sustainable agriculture that can satisfy the ever-rising global demand for food and animal feeds is becoming a manifest necessity in the light of the growing population, and the security concerns in the increasing land-use for food/feed. There has been a recent growth of interest in the positive impact of the functional ingredients derived from plants on human health. The proposed beneficial effects of such ingredients span from a decrease in plasma glucose levels and in preventing hypertension, to controlling obesity, improving satiety and stabilizing plasma lipid homeostasis. Lupin is a sustainable crop that plays an important role in organic farming, thus, the interest in lupin as a protein source for human and animal nutrition has increased in recent years.

The use of legumes as a source of protein is somewhat limited by the low digestibility of most plant proteins (Neves et al., 2006). Previous digestibility studies of protein obtained from legumes have shown interactions between antinutritional compounds, such as trypsin inhibitors and tannins, and the decreased proteolytic susceptibility of protein complexes, thereby decreasing the food value of plant proteins (Agte et al., 1998). The digestibility of lupin protein could be improved by lactic acid fermentation (Bartkiene et al., 2015).

However, it should be noted that lactic acid fermentation is a traditional process for food and feed production that may also result in the formation of undesirable compounds (e.g., biogenic amines and D-lactate). Also, lupin exhibits useful techno functional properties allowing its use as an ingredient in the production of several palatable food products, such as biscuits, pasta, and bread (Lee et al., 2006; Guillamon et al., 2010; Jayasena and Nasar-Abbas, 2011, 2012; Bartkiene et al., 2013a, 2016b). For instance, the supplementation of wheat flour with the high-protein lupin flours can improve the nutritional quality of baked goods (Gomez et al., 2008).

In addition, lupine does not contain gluten (a mixture of proteins found in wheat and related grains), thus, it could be used as a functional ingredient in gluten-free foods (Scarafoni et al., 2009). However, the use of lupin flour with a high protein content for the production of cereal products may cause problems associated with the formation of acrylamide (Bartkiene et al., 2013b, 2016a). The tolerable daily intake (TDI) levels of acrylamide for neurotoxicity were estimated to be 40 μg/kg per day and for cancer—2.6 μg/kg per day (Tardiff et al., 2010). Mitigation strategies propose modifying the product formulations or processing conditions, to minimize the pathways of acrylamide formation. For this reason, it is very important to assess the risk of new raw materials and technological procedures and to evaluate the safety parameters of the finished products.

## The application of lacto-fermentation to improve the nutritional and functional value of lupin

The *in vitro* digestibility of proteins depends on the type of fermentation (solid state or submerged fermentation), the type of microorganisms and the type of legume (Bartkiene et al., 2015). The low digestibility of plant proteins together with the limited content of essential amino acids result in a low nutritional value compared to animal proteins (Carbonaro et al., 2012). The fermentation of legumes enriches products with high-value proteins of microbial origin, reduces the concentration of antinutritional factors, increases the antioxidant activity, and generally improve the nutritional characteristics (Bartkiene et al., 2011; Curiel et al., 2015).

Fermentation with lactobacilli has been reported to increase the concentrations of free amino acids in legumes (Curiel et al., 2014; Coda et al., 2015). The fermentation method (solid state or submerged fermentation), type of microorganisms applied for the fermentation (lactic acid bacteria), and the variety of lupin significantly affect the content of free essential and nonessential amino acids in fermented lupin wholemeal (Bartkiene et al., 2016b). By optimizing the fermentation technology it is possible to produce bioactive peptides, which are of great interest for the design of functional foods and nutraceuticals.

Our previous study demonstrated the potential contribution of fermented lupin to the human diet through improving the gut environment and eliminating pathogenic bacteria (Bartkiene et al., 2013b). The effect of diet supplemented with lupin flour that was fermented with a probiotic strain of *Pediococcus acidilactici* on the gastrointestinal tract (GIT) of Wistar rats resulted in the enhanced activities of α-glucosidase, β-galactosidases, as well as high levels of lactic acid bacteria, bifidobacteria, and enterococci.

The lacto-fermentation of lupin flour had a significantly lowering effect on *Escherichia coli* compared to the control group. The dominant flora of the large intestine, like *Bifidobacterium* and anaerobic cocci, were found at high levels in diets containing fermented lupin.

## The safety parameters of fermented lupine—the concentrations of biogenic amines and D-lactic acid

Many strains of lactic acid bacteria have been referred to the European Food Safety Authority (EFSA) for safety assessment without raising any safety concerns. As a result, they have been included in the QPS (Qualified Presumption of Safety) list authorized for use in the food and feed chain within the European Union (EFSA, 2012). The same applies to the US, where they display the Generally Regarded as Safe (GRAS) status assigned by the U.S. Food and Drug Administration (FDA). However, some properties and enzymatic activity of the LAB can generate hazardous compounds such as biogenic amines (BAs), they should be avoided in food products (Linares et al., 2012).

Biogenic amines are formed by the enzymatic decarboxylation of amino acids and several factors as lupin variety, fermentation conditions, and the fermentative LAB strain have a significant effect on the free amino acid profile and content and, therefore, in the content of biogenic amines of lupins (Bartkiene et al., 2016b). Arginine is easily converted to agmatine and can be degraded to ornithine via bacterial activity, while ornithine undergoes decarboxylation to putrescine. Lysine can be converted by bacterial action into cadaverine. Histidine can be decarboxylated to histamine under certain conditions. Tyramine, tryptamine, and β-phenylethylamine arise in the same manner from tyrosine, tryptophan, and phenylalanine (Montet and Ray, 2016).

Microbially produced lactic acid is usually a mixture of the L(+)- and D(−)-forms. As the latter cannot be metabolized by humans, excessive intake can result in acidosis, which is a disturbance in the acid-alkali balance in the blood. The potential toxicity of D-lactic acid is of particular concern for malnourished and sick people (Motarjemi, 2002). The increased levels of D-lactate in plasma and urine have been demonstrated in cases of intestinal ischaemia, short bowel, and appendicitis, and are considered as a marker of dysbiosis and/or increased intestinal permeability (Verbeke et al., 2015). Therefore, the desirable lactic acid isomer that should be produced in food and feed fermentation is L-lactate.

## The application of lacto-fermentation to reduce acrylamide in bakery products supplemented with lupin flour

Cereals are deficient in lysine but are rich in cysteine and methionine. Legumes, on the other hand, are rich in lysine but deficient in sulfur-containing amino acids. Hereby, the overall protein quality could be improved by combining cereals with legumes. However, the addition of protein-rich legume flours could increase the content of acrylamide in baked products. The peptides and amino acids formed by protein breakdown may serve as precursors for acrylamide formation during the heating of foods containing reducing sugars. The elimination of acrylamide precursors using biotechnological tools is regarded as a promising way of eliminating acrylamide.

The reduction of acrylamide concentration in cereal products can be achieved by the prolonged fermentation of dough as a consequence of extensive asparagine consumption by yeast (Claus et al., 2008; Sadd et al., 2008). Lacto-fermentation decreased the concentrations of asparagine and reduced saccharides in lupin and thus reduced the acrylamide content in wheat-lupin biscuits (Bartkiene et al., 2016a). The levels of acrylamide in wheat-lupin bread could be lowered to 49.1% by fermentation with *L. sakei*. The fermentation of lupin sourdoughs with *Pedioccocus* strains can decrease the levels of acrylamide in bread by 18.7% on average, compared to bread made with untreated lupin flour.

For the purpose of enriching wheat bread with lupin as a high-quality protein source, pure lactic acid bacteria cultures, such as *L. sakei* and *P. pentosaceus*, characterized by higher proteolytic activity, has been recommended for the fermentation of lupin flour in sourdough production (Bartkiene et al., 2013c). The most effective acrylamide reduction in biscuits (by 85%) was reached using solid state fermentation of lupin with *P. acidilactici*. Fermented lupin supplementation offers a great potential in the development of bakery products with enhanced nutritional value and low acrylamide content.

## Conclusions

Lupin is a good alternative source of protein, enabling affordable nutritional enrichment of food and feed and providing better access to protein for underserved populations. Most of the research toward this goal has been focused on improving the antioxidant activity, sensory properties, and the functional and nutritional value of the products. In our opinion, the influence of new ingredients included in the food/feed formulas should be subject to more complex analysis. The formation of undesirable compounds during the manufacturing process is unavoidable, and specific technological solutions may be encouraged to mitigate these issues. Lactic acid fermentation with selected bacterial strains could be used for increasing the functional value of lupin.

A complex analysis during all technological steps should be performed to avoid the formation of toxic compounds, in order to ensure the safety of the food products. In this case, the appropriate technological parameters should be optimized (the selection of starters for fermentation and the choice of lupin variety, appropriate design of fermentation process).

## Author contributions

EB working on lupin proteins biotechnological treatment by using selected microorganisms, lupin proteins safety parameters and nutritional value evaluation. VB working on chemical methods development for toxic compounds in food produced with lupin seeds evaluation. VS working on lupin proteins isolation and safety parameters evaluation. DZ working on lupin proteins isolation and technological parameters evaluation. GJ working on lupin proteins hydrolysis and nutrition design.

### Conflict of interest statement

The authors declare that the research was conducted in the absence of any commercial or financial relationships that could be construed as a potential conflict of interest.
